# Development and Validation of the China Dietary Inflammatory Index (CHINA-DII)

**DOI:** 10.3390/nu17101687

**Published:** 2025-05-15

**Authors:** Yuhang Chen, Zhijie Luo, Lu Cheng, Qingying Wang, Fengqin Zou, Mohammad Abidullah Warsi, Yulan Lin

**Affiliations:** Department of Epidemiology and Health Statistics, Fujian Provincial Key Laboratory of Environment Factors and Cancer, School of Public Health, Fujian Medical University, Fuzhou 350122, China; yuhangchen@fjmu.edu.cn (Y.C.); zhijieluo@fjmu.edu.cn (Z.L.); lucheng@fjmu.edu.cn (L.C.); qingyingwang@fjmu.edu.cn (Q.W.); zfengq@fjmu.edu.cn (F.Z.); doctormaw@outlook.com (M.A.W.)

**Keywords:** gastric cancer, dietary inflammatory index, dietary nutrients, Chinese adults

## Abstract

**Objective:** To develop and validate the China Dietary Inflammatory Index (CHINA-DII) for Chinese adults. **Methods:** A systematic literature search was conducted to identify studies published between 2009 and 2024 reporting dietary intake levels among Chinese adults. After applying inclusion and exclusion criteria and evaluating study quality, a dietary intake database for Chinese adults was established. Following the methodology of the original Dietary Inflammatory Index (DII), the CHINA-DII was constructed and validated. A total of 256 newly diagnosed gastric cancer patients who visited the Union Hospital of Fujian Medical University between June 2023 and November 2024 were recruited. Demographic information, clinical data, and dietary data based on a food frequency questionnaire (FFQ) were collected. Spearman rank correlation was used to assess the relationship between CHINA-DII scores and high-sensitivity *C*-reactive protein (hs-CRP) levels. **Results:** A total of 33 eligible studies were included to develop a dietary intake database encompassing 27 dietary components. Among the 256 gastric cancer patients, the average CHINA-DII score was −1.91 ± 0.35, and the mean hs-CRP level was 3.68 ± 2.35 mg/L. CHINA-DII scores were positively correlated with hs-CRP levels (r = 0.20, *p* ≤ 0.001). Multivariate logistic regression analysis showed that individuals with higher CHINA-DII scores had a 1.90-fold increased risk of hs-CRP ≥ 3 mg/L compared to those with lower scores (odds ratio, OR = 1.90; 95% confidence interval, 95%CI: 1.01–3.55). For each 1-standard-deviation (SD) increase in CHINA-DII score, the risk of hs-CRP ≥ 3 mg/L increased by 1.50 times (OR = 1.50, 95% CI: 1.10–2.06). **Conclusions:** The CHINA-DII developed in this study effectively reflects the potential inflammatory impact of dietary intake in Chinese adults and is significantly positively associated with the inflammatory marker hs-CRP, indicating good validity and applicability.

## 1. Introduction

Inflammation is a protective physiological response triggered by the immune system in reaction to cellular and tissue damage. Its core mechanisms include the clearance of pathogens, repair of damaged tissues, and restoration of homeostasis, thereby effectively defending the host against infectious agents such as bacteria and viruses [1]. While a moderate inflammatory response is essential for maintaining health and homeostasis, dysregulation of the inflammatory process may lead to pathological inflammation [1]. Anti-inflammatory cytokines, such as interleukin-10 (IL-10), are integral components of a negative feedback system that helps suppress pro-inflammatory signaling pathways [2]. Failure of these regulatory mechanisms can result in the development of chronic systemic inflammation [2]. A hallmark of systemic inflammation is elevated levels of pro-inflammatory biomarkers in the bloodstream, including interleukin-1β (IL-1β), tumor necrosis factor-alpha (TNF-α), and *C*-reactive protein (CRP). This inflammatory response can be driven by both non-modifiable factors (e.g., hormonal changes, aging) and modifiable environmental factors (e.g., lifestyle, dietary patterns) [3]. Numerous studies have demonstrated that diet plays a critical role in modulating chronic inflammation in the body [4,5,6,7,8]. The Western dietary pattern, characterized by a high intake of red meat, processed foods, refined grains, and saturated fat-rich dairy products, has been positively associated with levels of inflammatory biomarkers. In contrast, the Mediterranean diet, rich in fruits, vegetables, fish, and olive oil, has been linked to a lower inflammatory status [4,5]. Certain nutrients, such as dietary fiber, vitamin C, and omega-3 fatty acids, are also associated with reduced inflammation [6,7,8].

In previous studies exploring the relationship between diet and inflammation, researchers often focused on individual food components, specific nutrients, or particular dietary patterns. However, the human body’s processes of digestion, absorption, and metabolism of nutrients and foods do not occur in isolation. Within complex dietary structures, the combination and interaction of various foods exhibit diverse characteristics, and the multiple nutrients present in these foods may have synergistic or antagonistic effects. Evidence suggests that evaluating single foods or nutrients alone cannot fully capture the comprehensive interactions within overall dietary intake. As a result, the academic community has shifted focus toward developing dietary indices or patterns that can holistically assess overall dietary quality. Methods for evaluating dietary quality can be broadly categorized into two approaches: a priori and a posteriori. The a priori approach is based on established evaluation systems and dietary guidelines, offering a direct and objective method of assessment [9]. Common a priori indices include the Diet Quality Index (DQI), Healthy Eating Index (HEI), and Diet Balance Index (DBI). In contrast, the a posteriori approach derives dietary assessment criteria from survey data through statistical methods [9], with common techniques including Partial Least Squares (PLS), cluster analysis, and factor analysis [10]. However, both a priori and a posteriori approaches lack the ability to evaluate the inflammatory potential of the diet.

The concept of the Dietary Inflammatory Index (DII) was first proposed in 2009 by a team led by epidemiologist Cavicchia at the University of South Carolina [11], and its assessment methodology was subsequently refined and optimized by Shivappa et al. in 2014 [12]. The updated DII incorporated 45 dietary components and utilized data from 11 food consumption databases across diverse global populations. Its calculation was based on a comprehensive review of the literature assessing the associations between these 45 dietary factors and 6 inflammatory biomarkers: IL-1β, IL-4, IL-6, IL-10, TNF-α, and *C*-reactive protein (CRP). Each dietary component was assigned a literature-derived inflammatory effect score, taking into account multiple factors such as study type, research design, dose–response relationships, and the strength and direction of associations with inflammatory markers [12]. These scores ranged from −1 to +1, with positive values indicating pro-inflammatory effects, negative values indicating anti-inflammatory effects, and zero representing no association with inflammation. The absolute magnitude of the score indicated the strength of the effect. Previous studies confirmed that the DII was significantly associated with various inflammation-related chronic health outcomes, including obesity [13], cancer [14], hypertension [15], type 2 diabetes [13,16], and atherosclerotic cardiovascular disease [17].

The reference intake database for the 45 dietary components used to calculate the DII included dietary data from 11 countries and regions worldwide [11]. The majority of these data were derived from Western countries, with relatively limited inclusion of dietary data from Asian regions, particularly China. Given China’s diverse dietary culture, its dietary structure differs significantly from that of Western countries. This difference is not only reflected in food composition, but may also manifest in variations in the types and amounts of nutrients consumed. Therefore, constructing an index specifically designed to assess the dietary inflammatory potential of Chinese adults (CHINA-DII) is of great importance.

This study aimed to establish a dietary intake database for Chinese adults by conducting an in-depth extraction of dietary data from China based on a literature review. The CHINA-DII calculation method will be developed, referencing the DII calculation method proposed by Shivappa et al. [12]. Additionally, hypersensitive *C*-reactive protein (hs-CRP) will be used as a biomarker to reflect individual inflammation levels, and the relationship between the CHINA-DII and hs-CRP will be analyzed to evaluate the effectiveness of the CHINA-DII. Serum hs-CRP levels have been widely used as a biomarker to evaluate the validity of the DII in previous studies. For example, a study from the Japan Cancer Screening Project reported partial correlation coefficients between the DII and hs-CRP of 0.064 in males and 0.012 in females [18]. A meta-analysis of 20 studies demonstrated a positive association between the DII and CRP (odds ratio, OR = 1.21; 95% confidence interval, 95% CI: 1.10–1.32) [19]. Similarly, a cross-sectional study from Korea observed that individuals in the highest quintile of DII scores had a 70% increased risk of elevated hs-CRP levels compared to those in the lowest quintile (OR = 1.70, 95% CI: 1.07–2.69) [20]. Moreover, a Japanese cohort study showed that each one-unit increase in the DII was associated with a 9% increase in the odds of elevated hs-CRP levels (OR = 1.09, 95% CI: 1.01–1.19) [21].

This new index aims to create an inflammation assessment tool more suitable for the diets of Chinese adults, improve disease prevention strategies based on general dietary guidelines, and enhance the effectiveness and specificity of relevant nutritional guidance.

## 2. Methods

### 2.1. Literature Search

This study conducted a systematic search in five databases: China National Knowledge Infrastructure (CNKI), Wanfang Data Knowledge Service Platform, VIP Chinese Science and Technology Journal Database, PubMed, and Web of Science. The main search keywords included the following: dietary, nutritional status, nutrition, intake, consumption, China, Chinese. The search time range for all databases was limited from 1 January 2009 to 31 December 2024.

The inclusion criteria for the literature were the following: (1) Chinese adults aged ≥18 years. (2) Any cross-sectional studies, cohort studies, case–control studies, etc., involving dietary intake in Chinese adults. For case–control studies, only data from the control group were collected. (3) Dietary assessment methods such as 24 h dietary recall (food diary, weighed food method, and food frequency questionnaires (FFQ). (4) The study must report data on dietary energy or nutrient intake in Chinese adults. The exclusion criteria were the following: (1) Participants: Non-Chinese adults or studies involving special conditions that may affect dietary intake, such as pregnant women, breastfeeding women, or individuals with diabetes. (2) Study types: Review abstracts, conference proceedings, letters, case reports. (3) Sample size < 200. (4) Surveys conducted before 2009. (5) Studies that did not provide dietary intake data or whose data could not be converted into mean and standard deviation forms. (6) Studies with duplicate publications from the same dataset, where only one study was retained. (7) Studies where the dietary assessment method had not been validated. (8) Studies with low quality.

### 2.2. Literature Screening and Quality Assessment

All of the literature was managed using EndNote X9 software, and duplicate references were deleted. The literature screening process was as follows: Two independent researchers reviewed the titles and abstracts of the literature to exclude studies that were clearly unrelated to the research topic. After the initial screening, the remaining literature was subjected to full-text review. Based on the pre-established inclusion and exclusion criteria, a final selection of the literature was made to determine the studies that met the research requirements.

This study first evaluated the quality of dietary intake survey methods. The primary dietary survey methods involved in the research included 24 h dietary recall, food diaries, the weighed food method, and FFQs. For studies using an FFQ, full-text literature on its validation study was retrieved. Specifically, if the FFQ had been validated, the study was included; if it had not been validated, it was excluded [22]. For studies using 24 h dietary recall, food diaries, or the weighed food method as dietary assessment tools, at least two days of dietary data were required for inclusion; otherwise, they were excluded [23].

The quality of the included studies was evaluated using the observational epidemiological research quality assessment criteria recommended by Cormick, G. et al. [24]. This standard includes 9 assessment dimensions: eligibility criteria, representativeness, reasonableness of data collection tools, response rate, clarity of study description, ethics, and consistency between research questions and reported data. Each dimension is scored 1 point, with evaluation standards categorized as “not mentioned or unsatisfactory” (0 points) and “satisfactory” (1 point). Each study was scored by dimension, with a total score range of 0–9. According to the Newcastle–Ottawa Scale (NOS) quality classification standard, studies with total scores of 1–3 were defined as low-quality studies, 4–6 as medium-quality studies, and ≥7 as high-quality studies [25]. Only medium-quality or higher studies were included for further analysis in this study.

The literature screening and quality assessment were independently completed by two researchers, strictly following the established inclusion and exclusion criteria, and cross-checked. In cases of discrepancies in evaluation opinions, the evaluators reached consensus through discussion and negotiation; if consensus could not be reached, a third-party expert was invited for discussion to ensure the objectivity and reliability of the assessment results. The evaluation criteria checklist is shown in Supplementary Table S1.

### 2.3. Data Extraction and Database Construction

After the full-text quality assessment, relevant data information was organized and extracted from the studies that met the inclusion criteria. The content extracted from the included studies included the following: (1) Basic information of the study: article title, first author, publication year. (2) Basic characteristics of the study: study period, study design type, sample size, study population region, inclusion and exclusion criteria for the study population, dropout and missing information, dietary survey method, types of nutrients, and detailed dietary intake.

In the process of extracting dietary intake data, this study used the following methods: For studies that reported sample size, median, maximum value, and minimum value, the method proposed by Hozo et al. [26] was used for extraction; for studies that reported sample size, median, upper quartile, and lower quartile, the method proposed by Wan et al. [27] was used for extraction; if a study divided the study population into multiple subgroups and only reported the sample size, mean, and standard deviation for each subgroup, the researchers used the method proposed by Liu Haining et al. [28] to integrate and calculate the data for each subgroup to obtain mean and standard deviation values. Finally, the mean and standard deviation of dietary intake data for Chinese adults were statistically analyzed. If the nutrient data came from more than three studies, the weighted mean (WM) and its 95% CI were calculated; otherwise, the average was directly calculated. The heterogeneity of the included studies was assessed using the inconsistency index (*I*^2^) and Cochran’s Q test.

### 2.4. Calculation of the China Dietary Inflammatory Index (CHINA-DII) Score

This study referred to the dietary component inflammatory potential scoring method proposed by Shivappa et al. [12]. The CHINA-DII calculation process followed the same procedure as the original DII developed by Shivappa et al. [12].

The calculation involved five steps, summarized as follows: (1) Dietary intake data of study participants were obtained through dietary questionnaire surveys. (2) For each dietary component, a *Z*-score representing individual exposure was calculated using the following formula: *Z* = (individual intake of a dietary component − the mean intake of that component from the Chinese adult dietary intake database)/standard deviation of intake from the Chinese database. (3) To reduce the influence of right-skewed distributions, the calculated *Z*-scores were then centralized and converted into percentile scores (*q*) ranging from −1 to +1, with 0 as the midpoint. (4) The dietary inflammatory index score for each dietary component was calculated as follows: CHINA-DII (individual component) = *q* × *i*, where “*i*” represents the literature-derived inflammatory effect score of the dietary component and “*q*” represents the centralized percentile value. (5) The total CHINA-DII score was obtained by summing the individual component-specific scores as follows: CHINA-DII = *i_1_* × *q_1_* + *i_2_* × *q_2_* + ⋯ + *i_n_* × *q_n_*. In this formula, *n* denotes the total number of dietary components included in the CHINA-DII calculation.

### 2.5. Study Design and Participants of Validation Study

The validation study adopted a cross-sectional design. Patients newly diagnosed with gastric cancer at Fujian Medical University Union Hospital between July 2023 and November 2024 were recruited. Gastric cancer patients were selected for this study because serum hs-CRP levels were available in this population, allowing for an initial validation of the CHINA-DII.

Inclusion criteria were as follows: (1) age ≥ 18 years; (2) newly diagnosed cases of gastric cancer confirmed by histopathological or cytological examination; (3) awareness of their diagnosis; (4) understanding of the study purpose and voluntary participation. Exclusion criteria were as follows: (1) missing more than 70 dietary items; (2) changes in dietary habits in the past year; (3) refusal to participate or uncooperative behavior during the survey; (4) cognitive dysfunction such as depression or unstable mental conditions; (5) language communication barriers; (6) implausible total energy intake (men: >4200 kcal or <800 kcal; women: >3500 kcal or <600 kcal); (7) other severe health conditions besides gastric cancer that may affect health status; (8) hs-CRP level greater than 10 mg/L.

### 2.6. Survey and Data Collection

This study collected the general demographic information of participants through face-to-face interviews, including age, gender, marital status, education level, occupation, monthly household income per capita, smoking, alcohol consumption, daily life stress level, etc. A self-designed FFQ was used to assess participants’ dietary intake. Clinical data were obtained through the hospital medical records system, including diagnostic information, date of diagnosis, hs-CRP levels, body mass index (BMI), Tumor–Node–Metastasis (TNM), and other relevant clinical indicators.

Patients were instructed to report their habitual dietary intake over the preceding 12 months when completing the FFQ. Specifically, the FFQ included 78 food items/food groups, categorized into 13 groups: staple foods, tubers, pickled/grilled foods, eggs, fresh meat, seafood, dairy products, snacks and nuts, beverages, legumes, fresh vegetables, fresh fruits, and dried foods. Nine frequency options were provided for food consumption: “≥4 times per day”, “2–3 times per day”, “once per day”, “4–6 times per week”, “2–3 times per week”, “once per week”, “1–3 times per month”, “occasionally”, and “never.” The FFQ’s reliability and validity were evaluated in a separate study among 152 healthy Chinese adults (manuscript under submission). The Spearman correlation coefficients for test–retest reliability ranged from 0.60 to 0.80 for food groups and 0.66 to 0.96 for nutrients, with intraclass correlation coefficients ranging from 0.53 to 0.91 and 0.57 to 0.97, respectively. Validity coefficients comparing FFQ and 3-day 24 h dietary recall ranged from 0.41 to 0.72 for food groups and 0.40 to 0.70 for nutrients. These results suggest that the FFQ has good reproducibility and acceptable validity for dietary assessment in the Fujian population.

Assessment of hs-CRP: On the morning of the day after admission, fasting serum samples were collected from patients and sent to the hospital’s clinical laboratory for analysis. The hs-CRP level was measured using an automated biochemical analyzer, with a normal reference range of 0–10 mg/L. According to the recommendations by the Centers for Disease Control and Prevention (CDC) and the American Heart Association (AHA) [29], hs-CRP concentrations are categorized into different risk levels: a concentration above 3 mg/L is considered to be significantly associated with increased disease risk.

### 2.7. Statistical Analysis

Continuous variables with a normal distribution are described using the mean (x^−^) and standard deviation (SD), while those not conforming to a normal distribution are described using the median (M) and interquartile range (P25, P75). Categorical variables are presented as frequency and percentage (*n*, %). The chi-square test was used to compare categorical variables between groups, while the *t*-test or analysis of variance (ANOVA) was used for continuous variables. Based on the median CHINA-DII score, the participants were divided into low-CHINA-DII and high-CHINA-DII groups. Spearman’s rank correlation coefficient was used to evaluate the correlation between CHINA-DII scores and hs-CRP levels. Logistic regression models were used to analyze the association between CHINA-DII groups and hs-CRP levels, calculating the odds ratio (OR) and corresponding 95% CI. All *p*-values were based on two-sided tests, and a significance level of 0.05 was considered statistically significant. Data analysis was conducted using the Statistical Package for the Social Sciences, version 26.0 (SPSS 26.0).

### 2.8. Ethical Considerations

This study was conducted in accordance with the principles of the Declaration of Helsinki and was approved by the Ethics Committee of Fujian Medical University (FJMU No. 2020[53]). Before participation, the purpose and content of the study were fully explained to the patients, and informed consent was obtained. Participants were free to withdraw from the study at any time if they experienced any discomfort, and refusal to participate had no impact on their medical care. All personal information of the participants was kept strictly confidential at all times.

## 3. Results

### 3.1. Literature Screening and Inclusion Results

**Figure 1:**
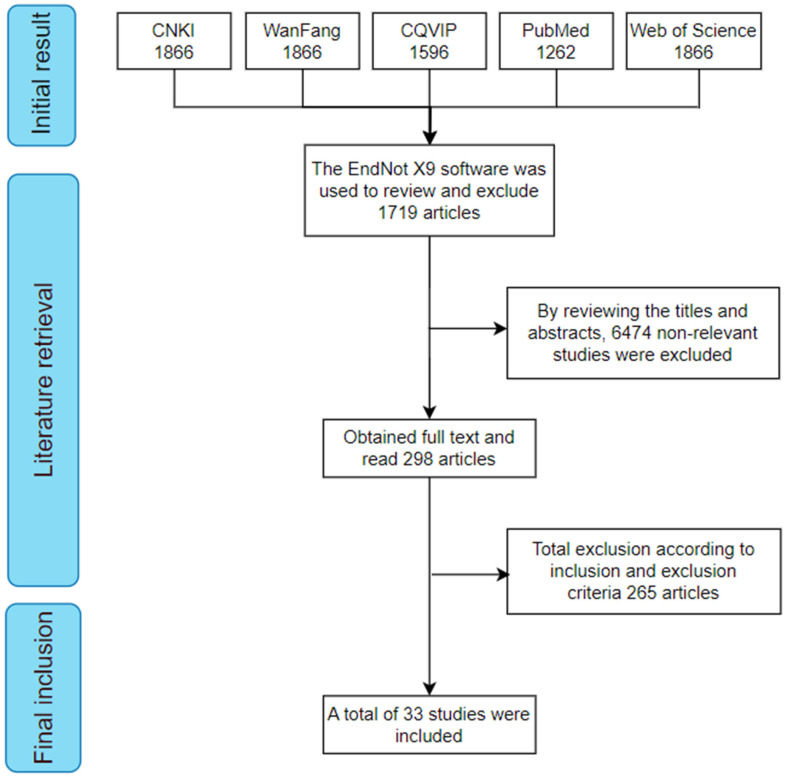
Flowchart of literature screening for meta-analysis.

A total of 8193 articles were identified based on the predefined search strategy. After importing all references into EndNote X9 for management, 1719 duplicate articles were removed, leaving 6474 articles. After preliminary screening (by reviewing titles and abstracts), 5824 articles that were clearly irrelevant to the research topic were excluded. The full texts of the remaining 298 articles were then reviewed, and 265 were excluded according to the inclusion and exclusion criteria. Ultimately, 33 articles met the criteria and were included in the study. The literature screening process is illustrated in Figure 1.

### 3.2. Quality Assessment of Included Studies

Based on the predefined quality assessment criteria, each of the 33 included studies was individually evaluated. All included studies had clearly defined research objectives, with response rates ≥70%, and demonstrated a high level of consistency between the research content and findings. Overall, a large proportion of the studies were of high quality, indicating a relatively low risk of bias. In summary, the quality and risk of bias of these 33 studies were considered acceptable (Table 1 and Table 2).

### 3.3. Characteristics of Included Studies

A total of 33 studies were included in this analysis, with cross-sectional studies accounting for the majority (75.8%). The number of dietary nutrients reported in these studies ranged from 1 to 21, including energy, protein, carbohydrates, fat, cholesterol, saturated fatty acids, monounsaturated fatty acids, polyunsaturated fatty acids, *n*-3 saturated fatty acids, *n*-6 saturated fatty acids, trans fatty acids, dietary fiber, folate, vitamin A, vitamin B1, vitamin B2, vitamin B3, vitamin B6, vitamin B12, β-carotene, vitamin C, vitamin D, vitamin E, iron (Fe), zinc (Zn), selenium (Se), and magnesium (Mg). The sample sizes of the included studies varied from a minimum of 200 participants to a maximum of 48,315 (Table 3).

### 3.4. CHINA-DII Dietary Intake Database for Chinese Adults

Among the 45 components originally used to establish the DII, some components were excluded from this study either due to a lack of relevant literature or because only frequency data were available without specific intake amounts. As a result, this study included 27 components from the DII to construct the CHINA-DII. Dietary components not included in the CHINA-DII calculation are listed in Supplementary Table S2. The literature sources and intake data for the CHINA-DII components are presented in Table 4.

### 3.5. Sociodemographic and Clinical Characteristics of the Study Participants for Validation

A total of 256 patients were included in the validation study, with a higher proportion of males (64.1%), resulting in a male-to-female ratio of approximately 2:1. The average age of the patients was 59.48 ± 10.91 years. The average CHINA-DII score for the patients was −1.91 ± 0.35, and the mean hs-CRP level was 3.68 ± 2.35 mg/L. Based on the median CHINA-DII score, the participants were divided into low-CHINA-DII (*n* = 128) and high-CHINA-DII groups (*n* = 128), and the demographic characteristics of the two groups were compared. The results showed that there were no statistically significant differences between the two groups in terms of age, marital status, education level, occupation, household per capita monthly income, daily life stress level, smoking, drinking, BMI, and TNM staging (*p* > 0.05). However, the proportion of males was higher in the high-CHINA-DII group (71.1%) compared to the low-CHINA-DII group (57.1%) (*p* = 0.019). See Table 5.

### 3.6. Dietary Nutrient Intake

The distribution of dietary nutrient intake in different CHINA-DII groups is shown in Table 6. Regarding the dietary nutrient intake, significant differences were observed between the two groups in the intake of energy, protein, carbohydrates, fat, cholesterol, dietary fiber, folate, vitamins (A, B1, B2, B3, B6, C, D, E), Zn, Mg, Fe, and Se (all *p* < 0.05).

### 3.7. Association Between CHINA-DII and hs-CRP

The Spearman correlation analysis between CHINA-DII score and hs-CRP indicated a positive association between CHINA-DII score and hs-CRP levels (r = 0.20, *p* < 0.05). Using a logistic regression model, with the low-CHINA-DII-score group as the reference, the association between the CHINA-DII and hs-CRP was explored, as shown in Table 7. In Model 1, without adjusting for other covariates, individuals in the high-CHINA-DII group had a 1.71 times higher risk of having hs-CRP ≥ 3 mg/L compared to those in the low-CHINA-DII group (OR = 1.71, 95% CI: 1.04–2.80), and for each 1 SD increase in CHINA-DII score, the risk of hs-CRP ≥ 3 mg/L increased by 1.40 times (OR = 1.40, 95% CI: 1.08–1.80). In Model 2, after adjusting for age group, gender, and BMI, the relationship between the CHINA-DII and hs-CRP levels was consistent with the unadjusted model. In Model 3, after further adjusting for other demographic variables, including education level, occupation, family per capita monthly income, daily life stress level, smoking, drinking, and TNM stage, the results were still consistent with the unadjusted model. Stratified analyses by gender and TNM stage were conducted, and the results are shown in Supplementary Tables S3 and S4. While similar trends were observed across subgroups, the associations were not statistically significant in some strata, likely due to the limited sample size within each stratum.

## 4. Discussion

This study constructed a dietary intake database for Chinese adults based on the existing literature and developed a China-specific DII following the principles of the original DII for assessing the inflammatory potential of dietary components. The CHINA-DII aims to accurately evaluate the inflammatory potential of diets in Chinese adults and serves as a powerful tool for further investigating the relationship between diet and health. The index was validated in the gastric cancer population, and the results demonstrated a positive association between CHINA-DII scores and the inflammation marker hs-CRP. This finding suggests that a higher CHINA-DII score (indicating more pro-inflammatory values) is associated with higher hs-CRP levels. Therefore, the CHINA-DII can be considered an effective tool for assessing the inflammatory potential of diets in Chinese adults. This dietary inflammation index-based evaluation system provides essential scientific evidence for future research, policy formulation, and dietary intervention measures.

### 4.1. CHINA-DII Database Source

The CHINA-DII is a dietary quality assessment tool based on the literature, designed to evaluate the overall inflammatory potential of an individual’s diet. A crucial step in its development was the establishment of a database that accurately reflects the dietary intake of Chinese adults. The construction of this database follows the same methodology as the original DII, which involves systematically searching the published literature on the dietary intake of Chinese adults and building a reference database following standardized procedures. The global dietary intake databases used for DII calculation predominantly rely on data from Western countries, with limited representation from Asian regions, especially China [12]. However, dietary habits in China significantly differ from those in Western countries. In this study, the CHINA-DII database for Chinese adults is based on 33 studies conducted across various age groups and regions, providing broad representativeness. Therefore, using the CHINA-DII to assess the dietary inflammatory potential of Chinese adults is more appropriate and accurately reflects the inflammatory potential of the Chinese population’s diet, offering a more reliable basis for related research and public health interventions.

Despite the broad representativeness of the CHINA-DII database, considerable heterogeneity (*I*^2^ > 99%) was observed across several nutrients. This heterogeneity likely arises from differences in study design, dietary assessment tools (e.g., FFQ vs. 24 h recall), regional dietary patterns, and the timeframes over which the included studies were conducted. Although a random-effects model was applied to account for between-study variability, this inherent variability may influence the robustness of the CHINA-DII reference database, particularly for nutrients with extreme heterogeneity (*I*^2^ > 99%). For example, it may lead to an over- or underestimation of the inflammatory potential of certain dietary components if the pooled values deviate significantly from the true central tendency. Therefore, the CHINA-DII should be interpreted as a population-level approximation that captures the average inflammatory potential of diet across diverse Chinese cohorts, rather than an exact individual-level measure. While the CHINA-DII provides an important tool tailored to Chinese dietary contexts, future refinements—such as stratified analyses by region or assessment method, or meta-regression to explore sources of heterogeneity—may help further improve the precision and applicability of this reference database for both research and public health planning.

### 4.2. Comparison of CHINA-DII and DII Nutrient Components

This study retained 27 dietary components out of the 45 originally identified from 1943 literature reviews to construct the CHINA-DII. This adjustment was primarily based on the differences in dietary characteristics between China and Western countries, as well as the existing research data on dietary intake among Chinese adults. The differences between this study and other DII scoring methods may be attributed to the lack of certain food parameters, especially anti-inflammatory food parameters. Some anti-inflammatory foods, such as turmeric, flavonoids, quercetin, saffron, flavan-3-ols, and onions, are generally consumed in small quantities, and thus may not significantly impact the scoring. Research indicates that the ability of the DII to predict dietary inflammatory potential does not significantly decrease when fewer than 30 dietary components are used [63,64], providing a methodological basis for the construction of the CHINA-DII.

In the comparison between Chinese and global dietary databases, it was found that Chinese adults consume lower amounts of protein, fat, and saturated fatty acids compared to the global average, while their intake of vitamin E was significantly higher. These differences are closely related to the traditional Chinese dietary structure, which is characterized by a predominance of plant-based fats, such as the use of canola and soybean oils that are rich in unsaturated fatty acids and vitamin E, leading to relatively lower fat intake. In contrast, Western diets are typically animal-protein-based, including beef, lamb, milk, and dairy products. These provide high-quality protein sources with amino acid compositions that are closer to human needs, and have higher biological availability, resulting in higher protein intake among Western populations.

### 4.3. Relationship Between CHINA-DII Scores and Inflammatory Markers

Inflammation is a defensive mechanism of the immune system against pathogens and injury, but chronic inflammation can cause damage to bodily tissues and organs [65]. Systemic inflammation is characterized by increased pro-inflammatory biomarkers in circulation. In this study, hs-CRP was used as the inflammatory marker to examine the relationship between CHINA-DII scores and inflammatory indicators. A positive correlation was found between hs-CRP levels and CHINA-DII scores, with a correlation coefficient of 0.20. Individuals in the high-score group (based on median split) had a 90% higher odds of hs-CRP ≥ 3 mg/L (OR = 1.90; 95% CI: 1.01–3.55) compared to the low-score group. This finding is consistent with previous studies on the relationship between the DII and inflammatory markers. For instance, an analysis using the original DII based on the 2015 Korean National Health and Nutrition Examination Survey (KNHANES) reported that individuals in the highest DII quintile had a 70% higher odds of elevated hs-CRP levels (>2 mg/L) compared to those in the lowest quintile (adjusted OR = 1.70; 95% CI: 1.07–2.69) [20]. These results indicate comparable or even stronger associations between diet and systemic inflammation in the Chinese population when using a region-specific index.

However, it still shall be noted that the validation of the CHINA-DII in this study relies solely on hs-CRP as a non-specific inflammatory marker. While hs-CRP is widely used and provides valuable insights into systemic inflammation, it may not fully capture the complexity of the inflammatory response. Inflammation involves a range of biomarkers, and hs-CRP alone may not account for all aspects of this process. Therefore, the incorporation or, at the very least, the discussion of other inflammatory markers, such as IL-6 and TNF-α, is important for further validating the CHINA-DII as an indicator of systemic inflammation. IL-6, for example, plays a central role in the regulation of immune responses and has been linked to chronic diseases, while TNF-α is a key pro-inflammatory cytokine involved in various inflammatory pathways. These markers, when included in future studies, could help provide a more comprehensive understanding of how the CHINA-DII reflects the broader inflammatory state in individuals.

### 4.4. Comparison of CHINA-DII and Other Dietary Quality Assessment Methods

Like other dietary patterns (such as the Healthy Eating Index and Mediterranean Diet Score), the CHINA-DII is a comprehensive evaluation tool based on the integration of a large amount of dietary information. This study showed a high degree of consistency in the relationship between dietary indices and inflammatory biomarkers with other dietary pattern studies. A meta-analysis on adult dietary patterns and their association with inflammatory biomarkers showed that the Mediterranean diet was associated with lower inflammation [66], which is also consistent with the systematic review by Barbaresko et al. from 2013 [67]. Another study from the European Prospective Investigation into Cancer and Nutrition (EPIC) found that long-term adherence to a globally sustainable healthy diet was mildly associated with reduced concentrations of inflammatory biomarkers, although the results were not statistically significant [68]. A prospective cohort study from the UK Biobank showed a negative correlation between the Healthy Eating Index, Mediterranean diet, and INFLA score (which integrates plasma inflammatory biomarkers such as white blood cell count, platelet count, *C*-reactive protein levels, and the neutrophil-to-lymphocyte ratio). For example, compared to participants in the lowest Mediterranean diet score category, those with Mediterranean diet scores of 4–5 and 6–9 had INFLA scores reduced by −0.31 (95% CI: −0.372, −0.244) and −0.555 (95% CI: −0.634, −0.475), respectively. A significant trend was also observed, where each 1 SD increase in the Mediterranean diet score and Healthy Eating Index was associated with a decrease in the INFLA score of −0.231 (95% CI: −0.260, −0.202) and −0.273 (95% CI: −0.302, −0.244), respectively [4]. A systematic review and meta-analysis on dietary patterns and chronic low-grade systemic inflammation in adults indicated that healthy dietary patterns were negatively correlated with inflammation markers (r = −0.13, 95% CI: −0.20,−0.06), whereas Western dietary patterns were positively correlated with inflammation markers (r = 0.11, 95% CI: 0.09, 0.12) [5].

Although the methods mentioned above have achieved a series of relatively consistent research conclusions in predicting inflammatory biomarkers, these research methods are essentially not designed to evaluate dietary quality from the perspective of inflammation potential. Unlike other dietary patterns or dietary indices, the core concept of the DII is to systematically evaluate dietary quality from the dimension of inflammation potential. Its development adopted a systematic scientific approach, involving an in-depth analysis and integration of approximately 2000 peer-reviewed articles, incorporating up to 45 different dietary parameters, thus creating a more comprehensive and precise dietary evaluation system [12]. This inflammation-potential-based evaluation method allows the DII to comprehensively reflect the potential impact of diet on inflammatory responses. Particularly in terms of predicting the risk of inflammation-related diseases, the DII may have advantages and greater accuracy compared to other traditional dietary evaluation methods. This makes the DII a more innovative and practically valuable tool for dietary quality assessment, providing new research perspectives and methodological support for the in-depth study of the relationship between diet, inflammation, and disease. Therefore, the CHINA-DII may be superior to other dietary evaluation methods in predicting diseases related to inflammation.

### 4.5. Limitations

This study also has certain limitations. First, the study participants in this research were limited to gastric cancer patients, and future studies shall be conducted in healthy populations to verify the generalizability of the results. Second, reverse causality and recall bias should be considered when interpreting the observed association between the CHINA-DII and hs-CRP levels in the current study. Although all participants were newly diagnosed gastric cancer patients and were instructed to report their habitual dietary intake over the past year, it is still possible that some individuals may have unconsciously altered their recollections or dietary patterns in response to early symptoms or the diagnosis itself. While this retrospective recall period helps mitigate the likelihood of capturing post-diagnosis dietary changes, recall bias remains an inherent limitation of FFQ-based dietary assessments, especially in clinical patients. Such bias may introduce the misclassification of dietary exposures and potentially attenuate or distort the true association. Future prospective studies with repeated dietary and biomarker assessments are needed to confirm the temporality and robustness of the association between the CHINA-DII and systemic inflammation. Third, only 27 of the original 45 DII components were included in the construction of the CHINA-DII due to the unavailability of corresponding intake data or relevant literature in the Chinese context. Although prior research suggests that the predictive capacity of the DII remains robust when fewer than 30 components are used, the exclusion of these components still represents a gap that should be addressed as more comprehensive dietary data become available. Finally, this study used only hs-CRP to verify the relationship between the CHINA-DII and inflammatory biomarkers. Future research could explore the relationship between the CHINA-DII and other inflammatory markers (such as interleukin-6, tumor necrosis factor-α, etc.) to assess its stability and consistency in inflammation evaluation. At the same time, exploring the association between the CHINA-DII and more inflammation-related diseases will provide more comprehensive theoretical support for disease prevention and treatment.

## 5. Conclusions

This study is the first to construct a CHINA-DII and to validate it in a population of gastric cancer patients. The results showed a significant positive correlation between CHINA-DII scores and hs-CRP levels. Based on these findings, the CHINA-DII developed in this study provides a scientific basis for accurately assessing the inflammatory potential of diets among Chinese adults. Furthermore, it offers a scientific evaluation tool for exploring the underlying mechanisms between diet and health.

## Figures and Tables

**Table 1:** Quality assessment of the studies included in the construction of the Chinese adult dietary intake database.

Study	Evaluation Item									Quality
	1	2	3	4	5	6	7	8	9	
Qin Qiulan [30]	1	1	1	1	1	1	1	1	0	High
Li Li [31]	1	1	1	1	1	1	1	1	1	High
Jia Xiaofang [32]	1	1	1	1	1	1	1	1	1	High
Linghu Liqin [33]	1	1	1	1	1	1	0.5	1	0	High
Han Xiaoli [34]	1	1	1	1	0	1	0.5	1	1	High
Chen Bingbing [35]	1	1	1	1	1	1	1	1	0	High
Zhao Fanglei [36]	1	1	1	1	0	1	1	1	1	High
Liu Ruru [37]	1	1	1	1	1	1	1	1	0	High
Pan Xin [38]	1	1	1	1	1	1	0	1	0	High
Tang Hongmei [39]	1	1	1	1	1	1	1	1	0	High
Song Pengkun [40]	1	1	1	1	0	1	1	1	1	High
Huang Qiumin [41]	1	1	1	1	1	1	1	1	1	High
Zhang Jianwen [42]	1	1	1	1	0	1	0.5	1	1	High
Chen Bingbing [43]	1	1	1	1	1	1	1	1	0	High
Zhang Yi [44]	1	1	1	1	1	1	0	1	1	High
Jin Wei [45]	0	1	0.5	1	0	1	0	1	1	Medium
Hou Liyuan [46]	0	1	1	1	0.5	1	0	1	1	Medium
Ma Liping [47]	1	1	1	1	0.5	1	0.5	1	1	High
Chen Chaogang [48]	1	1	1	1	0.5	1	0	1	1	High
He Denghua [49]	1	1	1	1	1	1	1	1	1	High
Zheng Xin [50]	0	1	1	1	0.5	1	0	1	0	Medium
Mo Baoqing [51]	0	1	1	1	1	1	0	1	1	High
Mo Baoqing [52]	0	1	1	1	1	1	0	1	1	High
Zhao-Min Liu [53]	1	1	1	1	0.5	1	1	1	1	High
Lianhong Li [54]	1	1	1	1	1	1	1	1	1	High
Rongping Zhao [55]	1	1	1	1	1	1	1	1	0	High
Zhuo Wang [56]	1	1	1	1	1	1	1	1	0	High
Chu-Yi Huang [57]	1	1	1	1	1	1	1	1	1	High
Xin Zhang [58]	1	1	1	1	1	1	1	1	1	High
Zhen Liu [59]	1	1	1	1	0	1	1	1	1	High
Hong Luo [60]	1	1	1	1	1	1	1	1	0	High
Rongge Qu [61]	1	1	1	1	1	1	1	1	1	High
Wen-qi Shi [62]	1	1	1	1	1	1	1	1	0	High

**Table 2:** Summary of the quality evaluation of the included studies in the construction of the Chinese adult dietary intake database.

Feature	Number of Studies (*n*)	Percentage (%)
Total	33	100
Inclusion/Exclusion criteria		
Clear	28	84.9
Unclear/Not clear	5	15.1
Loss to follow-up/Non-response		
Response rate ≥ 70%	33	100
Response rate < 70%	0	0
Not described/Not clear	0	0
Data collection		
Clearly defined and validated	32	97
Clearly defined but not validated	1	3
Outcome definition		
Clear	33	100
Unclear	0	0
Representativeness of study population		
Truly representative	22	66.7
Somewhat representative/Not clear	5	15.1
Specific population	6	18.2
Study purpose		
Clear	34	100
Not elaborated	0	0
Ethical approval and informed consent		
Both	21	63.7
One of the two	4	12.1
Not described	8	24.2
Consistency of study content and results		
Consistent	33	100
Not clear	0	0
Presentation of results		
Clear	20	60.6
Not clear	13	39.4
Quality evaluation grade		
High	30	90.9
Medium	3	9.1
Low	0	0

**Table 3:** Basic information of the studies included in the construction of the Chinese adult dietary intake database.

First Author/Survey Time	Publication Year	Study Type	Study Area	Sample Size	Dietary Assessment Method	Age (Years)	Types of Nutrients
Qin Qiulan [30] 2015–2017	2024	Cross-sectional	Guangxi province, China	2017	3 d 24 h weighing	18.4–98.1	13
Li Li [31] 2022–2023	2024	Cross-sectional	10 provinces in China	9981	3 d 24 h weighing	≥18	3
Jia Xiaofang [32] 2022–2023	2024	Cross-sectional	10 provinces in China	9364	3 d 24 h weighing	≥18	4
Linghu Liqin [33] 2020	2023	Cross-sectional	Shanxi province, China	391	7 d 24 h weighing	18–60	18
Han Xiaoli [34] 2017–2018	2023	Case–control	Urumqi city, China	450	3 d 24 h weighing	≥60	20
Chen Bingbing [35] 2015–2017	2023	Case–control	Nanping city, China	541	FFQ	18–70	2
Zhao Fanglei [36] 2015	2021	Cross-sectional	31 provinces in China	18,161	3 d 24 h weighing	≥65	4
Liu Ruru [37] 2010	2021	Cross-sectional	Shaanxi province, China	2241	FFQ	18–80	3
Pan Xin [38] 2017–2018	2020	Case–control	Binzhou city, China	441	FFQ	18–65	15
Tang Hongmei [39] 2012–2013	2019	Cross-sectional	Shanghai city, China	307	3 d 24 h weighing	≥18	11
Song Pengkun [40] 2010–2012	2019	Cross-sectional	National	16,621	3 d 24 h	≥60	4
Huang Qiumin [41] 2009, 2011, 2015	2019	Cross-sectional	9 provinces in China	19,076	3 d 24 h weighing	18–59	9
Zhang Jianwen [42] 2013–2017	2019	Cross-sectional	Linyi city, China	1795	3 d 24 h	≥60	12
Chen Bingbing [43] 2015–2017	2019	Case–control	Nanping city, China	546	FFQ	18–70	4
Zhang Yi [44] 2012–2013	2017	Cross-sectional	Leshan city, China	912	3 d 24 h	≥18	13
Jin Wei [45]	2017	Cross-sectional	Shanghai city, Jinan city, China	950	FFQ	≥60	13
Hou Liyuan [46]	2017	Case–control	Jiamusi city, China	214	SQFFQ	45–85	22
Ma Liping [47]	2015	Cross-sectional	Guangzhou city, China	553	FFQ	40–65	9
Chen Chaogang [48] 2011	2015	Cross-sectional	Guangzhou city, China	1382	FFQ	≥40	6
He Denghua [49] 2010–2012	2015	Cross-sectional	Hangzhou, Ningbo, Jinhua, Jiaxing, Huzhou, Lishui of China	1579	3 d 24 h weighing	≥18	16
Zheng Xin [50] 2009	2012	Cross-sectional	Liaoning province, China	536	3 d 24 h weighing	≥50	18
Mo Baoqing [51]	2011	Cross-sectional	Nangjing city, China	405	3 d 24 h weighing	35–55	20
Mo Baoqing [52]	2011	Cross-sectional	Benxi city, China	200	7 d 24 h weighing	35–55	21
Zhao-Min Liu [53] 2011–2017	2023	Cross-sectional	Guangzhou city, China	1987	FFQ	40–75	2
Lianhong Li [54] 2015–2015	2022	Cross-sectional	Guizhou province, China	899	FFQ	18–75	1
Rongping Zhao [55] 2015–2017	2022	Cross-sectional	31 provinces in China	48,315	3 d 24 h weighing	30–70	5
Zhuo Wang [56] 2020–2020	2021	Cross-sectional	Dingxi city, China	599	3 d 24 h weighing	≥18	14
Chu-Yi Huang [57] 2010–2019	2020	Case–control	Guangzhou city, China	2538	FFQ	30–75	5
Xin Zhang [58] 2010–2018	2020	Case–control	Guangzhou city, China	2389	FFQ	30–75	4
Zhen Liu [59] 2010–2012	2019	Cross-sectional	China national	16,621	3 d 24 h weighing	≥60	12
Hong Luo [60] 2010–2017	2019	Case–control	Guangzhou city, China	2144	FFQ	30–75	2
Rongge Qu [61] 2010	2018	Cross-sectional	Harbin city, China	6473	FFQ	20–75	6
Wen-qi Shi [62] 2011–2013	2015	Cross-sectional	Guangzhou city, China	3203	FFQ	40–75	3

Note: FFQ, food frequency questionnaire; SQFFQ, semi-quantitative food frequency questionnaire.

**Table 4:** Reference sources and mean and standard deviation of intake of each component in the CHINA-DII component database.

CHINA-DII Component	References (*n*)	Participants (*n*)	Reference Sources	*I* ^2^	WM (95% CI)	SD
Energy (kcal)	26	123,978	References [30,32,33,34,35,36,38,39,40,44,45,46,47,48,49,51,52,53,55,56,57,58,60,61,62]	99.88	1869.00 (1764.12,1973.88)	532.64
Protein (g)	20	66,004	References [30,32,33,34,36,38,39,40,42,44,45,46,47,48,49,51,52,56,61,62]	99.93	65.66 (61.20,70.11)	21.09
Carbohydrates (g)	18	62,494	References [30,32,33,34,36,38,40,42,44,45,46,47,48,49,51,52,56,61]	99.89	271.00 (248.01,293.98)	91.51
Fat (g)	20	113,258	References [30,32,33,34,36,38,40,42,44,45,46,47,48,49,51,52,55,56,58,61]	99.95	64.14 (54.87,73.41)	24.94
Cholesterol (mg)	7	5520	References [34,45,46,47,48,49,51]	97.31	328.19 (282.82,373.55)	194.62
Saturated fatty acids (g)	4	51,715	References [37,43,47,55]	99.41	18.42 (11.94,24.91)	8.60
Monounsaturated fatty acids (g)	4	51,715	References [37,43,47,55]	99.87	30.74 (21.93,39.55)	12.01
Polyunsaturated fatty acids (g)	4	51,715	References [37,43,47,55]	99.85	19.04 (14.39,23.69)	6.00
Dietary fiber (g)	13	17,952	References [30,34,38,44,45,46,47,48,49,51,52,58,61]	99.86	12.42 (9.50,15.35)	5.88
Folate (μg)	4	20,000	References [33,34,57,59]	99.98	139.20 (35.60,242.81)	58.22
Vitamin A (μgRE)	13	26,605	References [30,33,34,38,39,42,44,45,46,49,50,51,59]	98.84	376.13 (314.91,437.35)	294.38
Vitamin B1 (mg)	15	46,071	References [30,33,34,38,39,41,42,44,45,46,49,50,52,56,59]	100	0.92 (0.86,0.98)	0.31
Vitamin B2 (mg)	15	46,814	References [30,33,34,38,39,41,44,45,46,49,50,52,56,57,59]	99.99	0.81 (0.72,0.89)	0.31
Vitamin B3 (mg)	10	25,503	References [30,33,34,38,41,46,49,50,52,56]	99.99	13.31 (12.15,14.48)	3.56
Vitamin B6 (mg)	4	20,208	References [34,56,57,59]	99.96	0.51 (0.09,0.94)	0.27
Vitamin B12 (μg)	4	19,823	References [33,46,57,59]	99.96	0.98 (−0.41,2.37)	0.84
Vitamin C (mg)	16	46,480	References [30,33,34,38,39,41,42,44,45,46,49,50,51,52,56,59]	100	80.35 (72.24,88.47)	35.90
Vitamin D (μg)	3	4826	References [34,53,58]	99.87	3.74 (−2.87,10.35)	2.93
Vitamin E (mg)	16	48,771	References [30,33,34,38,39,41,42,45,46,49,50,51,52,56,59,62]	100	26.31 (20.89,31.73)	11.26
Zn (mg)	16	55,925	References [30,31,33,34,38,39,41,42,44,45,46,49,51,52,56,59]	100	9.67 (8.95,10.39)	2.96
Mg (mg)	13	42,994	References [33,34,38,39,41,42,44,46,49,51,52,56,59]	100	280.13 (264.72,295.53)	72.59
Fe (mg)	17	58,069	References [30,31,33,34,38,39,41,42,44,45,46,49,51,52,56,59,60]	99.99	19.01 (17.68,20.34)	6.34
Se (μg)	14	52,975	References [31,33,34,38,39,41,42,44,46,49,51,52,56,59]	99.98	42.91 (40.08,45.74)	16.03

Note: *I*^2^, inconsistency index; WM: weighted mean; SD, standard deviation.

**Table 5:** Sociodemographic and clinical characteristics of the study population (*n* = 256).

Variable	Total (*n* = 256)	Low CHINA-DII (*n* = 128)	High CHINA-DII (*n* = 128)	*p*-Value
Age (years)	59.48 ± 10.91	59.63 ± 10.99	59.34 ± 10.86	0.833
Age group (years)				
≤60	122 (47.7)	64 (50.0)	58 (45.3)	0.453
>60	134 (52.3)	64 (50.0)	70 (54.7)	
Sex				0.019
Male	164 (64.1)	73 (57.0)	91 (71.1)	
Female	92 (35.9)	55 (43.0)	37 (28.9)	
Marital status				0.281
Married	248 (96.9)	126 (98.4)	12 2(95.3)	
Unmarried/Separated/Divorced/Widowed	8 (3.1)	2 (1.6)	6 (4.7)	
Education level				0.598
Primary school or below	111 (43.4)	54 (42.2)	57 (44.5)	
Secondary school	69 (27.0)	34 (26.6)	35 (27.3)	
High school/Vocational high school	38 (14.8)	22 (17.2)	16 (12.5)	
College	17 (6.6)	10 (7.8)	7 (5.5)	
University	21 (8.2)	8 (6.2)	13 (10.2)	
Occupation				0.614
Farmers/Workers/Manual laborers	72 (28.1)	38 (29.7)	34 (26.6)	
Housewives/Retired/Unemployed	111 (43.4)	57 (44.5)	54 (42.2)	
Other occupations	73 (28.5)	33 (25.8)	40 (31.2)	
Family month income (RMB)				0.349
<3000	21 (8.2)	9 (7.0)	12 (9.4)	
3000–6000	101 (39.5)	56 (43.8)	45 (35.2)	
>6000	134 (52.3)	63 (49.2)	71 (55.4)	
Smoking				0.372
Yes	103 (40.2)	48 (37.5)	55 (43.0)	
No	153 (59.8)	80 (62.5)	73 (57.0)	
Alcohol drinking				0.500
Yes	42 (16.4)	19 (14.8)	23 (18.0)	
No	214 (83.6)	109 (85.2)	105 (82.0)	
Daily life stress				0.333
High/Medium	73 (28.5)	40 (31.3)	33 (25.8)	
Low/None	183 (71.5)	88 (68.7)	95 (74.2)	
BMI (kg/m^2^)				0.699
<24	159 (62.1)	78 (60.9)	81 (63.3)	
≥24	97 (37.9)	50 (39.1)	47 (36.7)	
TNM				0.373
I	86 (33.6)	42 (32.8)	44 (34.4)	
II	42 (16.4)	26 (20.3)	16 (12.5)	
III	71 (27.7)	33 (25.8)	38 (29.7)	
IV	4 (1.6)	3 (2.3)	1 (0.8)	
Missing	53 (20.7)	24 (18.8)	29 (22.7)	
hs-CRP (mg/L)	3.68 ± 2.35	3.38 ± 2.25	3.98 ± 2.42	0.041
CHINA-DII	−1.91 ± 0.35	−2.40 ± 0.41	−1.42 ± 0.39	<0.001

Note: Continuous variables are presented as mean (SD) and compared using the *t*-test; categorical variables are shown as frequencies (%) and compared using Chi-square test; abbreviation: RMB, Renminbi (Chinese currency); BMI, body mass index; hs-CRP: high-sensitivity *C*-reactive protein; CHINA-DII: China Dietary Inflammatory Index.

**Table 6:** Comparison of nutrient intake across gastric cancer patients with varying CHINA-DII scores.

Nutrients	Total (*n* = 256)	Low CHINA-DII (*n* = 128)	High CHINA-DII (*n* = 128)	*p*-Value
Energy (kcal)	1609.7 ± 551.74	1883.02 ± 554.25	1336.56 ± 391.87	<0.001
Protein (g)	83.23 ± 36.24	101.44 ± 38.05	65.02 ± 22.82	<0.001
Carbohydrates (g)	209.35 ± 72.51	241.06 ± 73.55	177.64 ± 55.86	<0.001
Fat (g)	52.70 ± 25.52	62.42 ± 27.33	42.97 ± 19.24	<0.001
Cholesterol (mg)	548.90 ± 306.32	673.72 ± 333.69	424.08 ± 213.64	<0.001
Dietary fiber (g)	11.48 ± 73.57	14.86 ± 6.26	8.09 ± 2.84	<0.001
Folate (μg)	157.98 ± 73.57	196.85 ± 72.06	119.11 ± 51.27	<0.001
Vitamin A (μgRE)	574.17 ± 259.90	722.70 ± 261.46	425.64 ± 150.98	<0.001
Vitamin B1 (mg)	0.74 ± 0.31	0.90 ± 0.32	0.57 ± 0.19	<0.001
Vitamin B2 (mg)	1.05 ± 0.41	1.28 ± 0.43	0.82 ± 0.24	<0.001
Vitamin B3 (mg)	20.41 ± 7.49	23.82 ± 7.59	17.01 ± 5.62	<0.001
Vitamin B6 (mg)	0.32 ± 0.22	0.43 ± 0.24	0.21 ± 0.12	<0.001
Vitamin C (mg)	117.21 ± 86.24	147.38 ± 94.23	87.04 ± 64.96	<0.001
Vitamin D (μg)	2.26 ± 1.40	2.80 ± 1.55	1.73 ± 0.97	<0.001
Vitamin E (mg)	11.27 ± 6.14	14.44 ± 6.49	8.11 ± 3.68	<0.001
Zn (mg)	15.89 ± 5.10	18.69 ± 5.13	13.10 ± 3.17	<0.001
Mg (mg)	74.62 ± 41.85	94.89 ± 45.76	54.34 ± 24.36	<0.001
Fe (mg)	21.67 ± 7.41	26.02 ± 7.33	17.32 ± 4.28	<0.001
Se (μg)	330.96 ± 117.44	406.61 ± 112.60	255.31 ± 58.96	<0.001

Note: Continuous variables are presented as mean (SD) and compared using the *t*-test.

**Table 7:** Logistic regression for the association between CHINA-DII and hs-CRP.

Model	Low CHINA-DII	High CHINA-DII	*p*-Value	per SD Increase	*p*-Value
hs-CRP					
Model 1	1.00	1.71 (1.04–2.80)	0.034	1.40 (1.08–1.80)	0.010
Model 2	1.00	1.69 (1.02–2.81)	0.044	1.40 (1.08–1.82)	0.011
Model 3	1.00	1.90 (1.01–3.55)	0.046	1.50 (1.10–2.06)	0.011

Note: Logistic regression models were as follows: Model 1, unadjusted; Model 2, adjusted for age group, sex, and BMI; Model 3, further adjusted for education level, occupation, monthly household income per capita, daily life stress level, smoking, alcohol consumption, and TNM stage based on Model 2.

## Data Availability

The data that support the findings of this study are available from the corresponding author, Yulan Lin, upon reasonable request. The data are not publicly available due to privacy and ethical restrictions.

## Supplementary Materials

The following supporting information can be downloaded at: https://www.mdpi.com/article/10.3390/nu17101687/s1, Supplementary Table S1. List of methodological quality assessment scales included in the literature. Supplementary Table S2. Dietary components not included in CHINA-DII. Supplementary Table S3. Spearman correlation between CHINA-DII and hs-CRP stratified by sex and TNM stage. Supplementary Table S4. Logistric regression for the association between CHINA-DII and hs-CRP stratified by sex and TNM stage.

## Abbreviations

CHINA-DII	China Dietary Inflammatory Index
DII	Dietary Inflammatory Index
PLS	Partial Least Squares
FFQ	Food frequency questionnaire
hs-CRP	High-sensitivity *C*-reactive protein
OR	Odds ratio
95%CI	95% confidence interval
DQI	Diet Quality Index
HEI	Healthy Eating Index
DBI	Diet Balance Index
CNKI	China National Knowledge Infrastructure
WM	Weighted mean
*I* ^2^	Inconsistency index
BMI	Body mass index
CDC	Centers for Disease Control and Prevention
AHA	American Heart Association
SD	Standard deviation
M	Median
ANOVA	Analysis of variance
