# Supplementary material for: Development and Validation of the China Dietary Inflammatory Index (CHINA-DII)
Source: Nutrients. 2025 May 15;17(10):1687. doi: 10.3390/nu17101687 (PMC12114556; doi:10.3390/nu17101687)
Supplement: Supplementary file 1 [file nutrients-17-01687-s001.zip › nutrients-3622686-supplementary.pdf]

Supplementale Table S1 List of methodological quality assessment scales included in the literature

Item	Evaluation criteria
Inclusion and exclusion criteria (1 point)	①The population definition is clear and specific (1 point)<br>②The population definition is unclear (0 points)
Loss to follow-up or non-response (1 point)	①The characteristics of the study subjects and non-respondents are well comparable, and the response rate >70% (1 point)<br>②The characteristics of the study subjects and non-respondents are less comparable, and the response rate <70% (0.5 points)<br>③The comparability of characteristics between study subjects and non-respondents is not clearly defined, and the response rate is not reported (0 points)
Data collection (1 point)	①The assessment tools are clearly defined with information on their development or source (1 point)<br>②The assessment tools are clearly defined but lack information on their development or source (0.5 points)<br>③The assessment tools are described vaguely or incompletely (0 points)
Outcome definition (1 point)	①The outcome is clearly defined (1 point)<br>②The outcome is unclear or ambiguous (0 points)
Representativeness of study population (1 point)	①The sample fully represents the target population (e.g., complete population or random sampling) (1 point)<br>②The sample partially represents the target population (e.g., non-random sampling) or the sampling method is not clearly explained (0.5 points)<br>③Only specific categories of the population are selected (0 points)
Research objective (1 point)	①The objective is clearly stated (1 point)<br>②The objective is poorly stated (0.5 points)<br>③The objective is not clearly explained (0 points)
Ethical considerations (1 point)	①Informed consent is obtained from the study subjects and ethical approval is granted (1 point)<br>②Informed consent or ethical approval is not explicitly reported (0.5 points)<br>③Informed consent is not explicitly reported, and ethical approval is not mentioned (0 points)
Clarity of data presentation (1 point)	①Data presentation is clear, with complete reporting of numerators, denominators, and missing values (1 point)<br>②Data presentation is unclear, with unreported or vague descriptions (0 points)
Consistency (1 point)	①The study content and data reporting are consistent (1 point)<br>②The study content and data reporting have some discrepancies (0 points)

Supplementale Table S2 Dietary components not included in CHINA-DII

Dietary components	Overall inflammatory potential score	Global daily intake (daily)	SD
Alcohol (g)	–0.278	13.98	3.72
Caffeine (g)	–0.110	8.05	6.67
Green tea/Black tea (g)	–0.536	1.69	1.53
Ginger (g)	–0.453	59.00	63.20
Garlic (g)	–0.412	4.35	2.90
Onion (g)	–0.301	35.90	18.40
Chili (g)	–0.131	10.00	7.07
Thyme/oregano (mg)	–0.102	0.33	0.99
Rosemary (mg)	–0.013	1.00	15.00
Flavan-3-ol (mg)	–0.415	95.80	85.9
Flavonol (mg)	–0.467	17.70	6.79
Anthocyanin (mg)	–0.131	18.05	21.14
Eugenol (mg)	–0.140	0.01	0.08
Soy isoflavones (mg)	–0.593	1.20	0.20
Saffron (g)	–0.140	0.37	1.78
Turmeric (mg)	–0.785	533.60	754.30
Flavonoids (mg)	–0.616	1.55	0.07
Flavanone (mg)	–0.250	11.70	3.82

Supplementale Table S3 Spearman correlation between CHINA-DII and hs-CRP stratified by sex and TNM stage.

Subgroups	*r*	*P* value
Sex		
Male	0.14	0.071
Female	0.28	0.007
TNM		
I/II	0.24	0.007
III/IV	0.10	0.401

Supplementale Table S4 Logistic regression for the association between CHINA-DII and hs-CRP stratified by sex and TNM stage.

Subgroups	hs-CRP≥3mg/L/hs-CRP<3mg/L	Low CHINA-DII	High CHINA-DII	*P value*
Sex				
Male	76/88	1.00	2.12 (0.92-4.85)	0.077
Female	53/39	1.00	2.31 (0.65-8.18)	0.196
TNM				
I/II	68/60	1.00	2.08 (0.92-4.70)	0.077
III/IV	40/35	1.00	2.17 (0.52-9.09)	0.287

Note: The logistic regression model was adjusted for age group, sex, BMI, marital status, education level, occupation, monthly household income per capita, daily life stress level, smoking, and alcohol consumption (excluding the stratification factor).
